# Transient Neonatal Myasthenia Gravis in a Newborn Infant Presenting to an Emergency Department

**DOI:** 10.7759/cureus.112517

**Published:** 2026-07-12

**Authors:** Marice Salib, Curtis Knight, Tommy Y Kim

**Affiliations:** 1 Department of Emergency Medicine, HCA Healthcare, Riverside Community Hospital, Riverside, USA

**Keywords:** acetylcholine receptor antibody, myasthenia gravis, newborn, poor feeding, transient

## Abstract

A four-day-old female born full term via uncomplicated vaginal delivery presented to the emergency department with poor oral intake. There was a significant maternal history of myasthenia gravis requiring pyridostigmine and intravenous immunoglobulin infusions five days per month. It was suspected that the patient had transient maternal acetylcholine antibodies causing the weakness and poor oral intake. The patient was admitted to the neonatal intensive care unit for further evaluation and management. Laboratory evaluation revealed elevated acetylcholine receptor antibodies at 2.69 nmol/L, which confirmed transient transfer of maternal antibodies. After a six-day hospitalization, the patient had improved oral intake, appropriate weight gain, and resolution of her weakness. The patient was safely discharged home.

## Introduction

Transient neonatal myasthenia gravis (TNMG) is a rarely encountered condition seen in the emergency department (ED) caused by the transfer of maternal acetylcholine receptor (AChR) antibodies across the placenta, resulting in neuromuscular weakness in newborns [1]. TNMG requires early recognition and intervention given the potential consequences including respiratory compromise, severe feeding difficulties, and, in rare cases, arthrogryposis multiplex congenita (AMC) caused by intrauterine antibody exposure [2,3]. TNMG is reported to affect 10-20% of infants born to mothers with myasthenia gravis (MG), but this likely underestimates the true incidence, as recent nationwide data found that up to 40% of MG-exposed infants required neonatal care or demonstrated feeding difficulties and hypotonia [4,5]. This case illustrates the subtle clinical findings and management strategies of an infant presenting to the ED with symptoms suggestive of TNMG.

## Case presentation

A four-day-old female presented to the ED with poor oral intake and decreased wet diapers. She was born at full term following an uncomplicated vaginal delivery. The patient’s mother had prenatal care from 10 weeks’ gestation. The maternal past medical history was notable for a six-year history of MG and maintenance on pyridostigmine and five days of intravenous immunoglobulin (IVIG) infusions every month. The patient had a healthy sibling with no history of similar episodes at the time of their birth or early infancy. On initial evaluation, the patient was tachycardic with a heart rate of 187 beats per minute; otherwise, the remaining vital signs were within normal limits for age. On examination, the infant had a weak cry but demonstrated a good baseline tone for age. The patient was also noted to have significant examination findings of a persistently open mouth with dry mucous membranes.

The differential for poor feeding in a neonate was considered, and the workup included laboratory tests with a complete blood count and a complete metabolic panel. Laboratory tests that were slightly outside the normal range were considered clinically not significant to the infant's presentation (Table 1). Due to the maternal history of MG and the patient’s presentation, TNMG was suspected as the possible etiology of the poor feeding and an AChR antibody test was also ordered. Due to the risk of developing respiratory difficulties given the nature of her suspected underlying TNMG, the patient was admitted to the neonatal intensive care unit (NICU) for close monitoring and supportive care. Laboratory evaluation revealed an elevated AChR antibody level at 2.69 nmol/L (Table 1), confirming passive transfer from maternal circulation. The markedly elevated level, over 10 times the upper limit of normal, established the diagnosis of TNMG.

**Table 1:** Laboratory tests WBC: white blood cell; RBC: red blood cell; Hgb: hemoglobin; Hct: hematocrit; MCV: mean corpuscular volume; MCH: mean corpuscular hemoglobin; MCHC: mean corpuscular hemoglobin concentration; RDW: red blood cell distribution width; Plt: platelets; MPV: mean platelet volume; BUN: blood urea nitrogen; AST: aspartate aminotransferase; ALT: alanine aminotransferase; ALP: alkaline phosphatase; AChR: acetylcholine receptor

Laboratory tests	Result	Reference range	Units
WBC	15.9	5.0 – 15.0	K/mm^3^
RBC	4.97	3.6 – 5.0	M/mm^3^
Hgb	10.9	10.0 – 15.0	gm/dL
Hct	35.0	30.0 – 45.0	%
MCV	70.4	80.0 – 99.0	fL
MCH	21.9	24.0 – 30.0	pg
MCHC	31.1	32.0 – 37.5	g/dL
RDW	14.6	11.0 – 14.5	%
Plt count	552	150 – 400	K/mm^3^
MPV	9.6	9.4 – 12.4	FL
Sodium	140	136 – 145	mmol/L
Potassium	3.9	3.5 – 5.1	mmol/L
Chloride	113	98 – 107	mmol/L
Carbon Dioxide	18	21 – 32	mmol/L
Glucose	158	74 – 106	mg/dL
BUN	4	7 – 18	mg/dL
Creatinine	0.17	0.70 – 1.30	mg/dL
Total protein	6.9	6.4 – 8.2	g/dL
Albumin	4.1	3.4 – 5.0	g/dL
Calcium	9.6	8.5 – 10.1	mg/dL
Total bilirubin	0.4	0.2 – 1.0	mg/dL
AST	67	15 – 37	U/L
ALT	40	12 – 78	U/L
Total ALP	300	45 – 117	U/L
Lipase	46	13 – 75	U/L
AChR antibody	2.69	0 – 0.24	nmol/L

In the NICU, continuous cardiorespiratory monitoring was initiated to monitor and intervene for respiratory distress if the need arose. There were no respiratory complications during her hospitalization. Feedings using a nasogastric tube were initiated while advancing her oral diet to ensure adequate caloric intake during the period of neuromuscular weakness. During the hospitalization, she was able to demonstrate clinical improvement, advancing to full oral feedings with demonstration of adequate weight gain. The patient was discharged home at 10 days of age after a six-day NICU hospitalization.

## Discussion

This case report highlights the clinical presentation, diagnosis, and management of TNMG secondary to maternal MG. The clinical presentation of TNMG is variable, ranging from subtle feeding difficulties that may initially go unnoticed to severe respiratory weakness requiring mechanical ventilation [5]. In a combined case series of 147 infants with TNMG, Kochhar et al. reported that only one infant required intubation for hypoventilation [6]. The examination of this patient was significant for a persistently open mouth, which was consistent with facial and bulbar muscle weakness characteristic of TNMG [3]. This infant also presented at four days of age, consistent with published data on TNMG onset. While symptoms often develop within hours after birth, they may be delayed for 24 hours or longer [7]. Data suggest that routine postnatal observation for at least four days is appropriate for infants born to mothers with MG, as the mean onset of myasthenic signs is 1.5 ± 2.6 days after birth [6]. Although this patient did not develop respiratory distress, respiratory compromise remains the most serious potential complication of TNMG and is the primary reason for NICU-level monitoring [5,8]. Current U.K. best practice guidelines recommend that newborn babies born to mothers with MG should have rapid access to neonatal high-dependency support, even if the mother's MG is well-controlled [8]. Although pharmacological treatment with acetylcholinesterase inhibitors or IVIG can be considered in neonates with more severe symptoms, most infants do well with observation and supportive care alone [5,9]. Given the mild-to-moderate severity of this patient's presentation and the self-limiting nature of TNMG, pharmacologic intervention was not required.

Due to the unpredictable nature of TNMG, vigilant monitoring is recommended for all infants born to mothers with MG, regardless of maternal disease severity or antibody titers [2]. The severity of neonatal disease has been shown to correlate with maternal AChR antibody concentrations, particularly antibodies directed against the fetal AChR γ subunit [3]. However, the occurrence of TNMG does not consistently correlate with maternal disease severity or antibody concentration. This variability is likely attributable to differences in AChR epitopes, epitope-binding affinity, and other non-AChR factors [2]. The mother in this case was receiving monthly IVIG, which is considered safe during pregnancy and may reduce the risk of severe fetal complications [2,9,10]. One small study of five pregnant women with MG treated with monthly IVIG as monotherapy reported no cases of TNMG [11]. However, as this case demonstrates, IVIG does not eliminate the risk of TNMG.

In this infant, early recognition and multidisciplinary management in the NICU resulted in a favorable short-term outcome with improved feeding tolerance and weight gain. The diagnosis in this case hinged on obtaining a thorough maternal medical history. This case underscores that collecting an extensive history regarding maternal medical problems and medications can provide crucial information leading to proper diagnosis and management. TNMG can occur even when the mother is symptom-free, making it essential to screen for maternal MG in any neonate with unexplained hypotonia or feeding difficulties [1]. Recent evidence suggests that TNMG may be underdiagnosed, with up to 40% of infants born to mothers with MG demonstrating feeding difficulties and hypotonia or requiring neonatal care [4]. The differential diagnosis for a neonate presenting with hypotonia and feeding difficulties is broad, including congenital myasthenic syndromes, congenital myopathies, spinal muscular atrophy, metabolic disorders, and central nervous system pathology.

TNMG is a self-limiting disease characterized by weakness that persists only while maternal antibodies remain in the neonatal circulation, with a mean duration of approximately 2-3 weeks [5]. Approximately 90% of infants with TNMG have complete resolution of symptoms within two months, and the remaining 10% recover within 4 months [5]. When symptoms persist beyond four months, consideration should be given for alternate diagnosis related to maternal MG, including disorders within the spectrum of fetal acetylcholine receptor antibody-related disorders (FARAD) [12]. FARAD encompasses a spectrum of disorders ranging from the relatively less severe permanent myopathic phenotype known as fetal AChR inactivation syndrome to the more severe often lethal AMC [12]. Fortunately, neither of these disorders was present in this patient. However, infants born to mothers with MG carry at least a 10-fold increased risk of developing MG or other autoimmune diseases later in life, warranting long-term awareness and monitoring [9]. Outpatient follow-up is essential to monitor developmental milestones, feeding, and the complete resolution of neuromuscular symptoms.

Long-term follow-up is crucial to assess developmental milestones and mitigate potential neurodevelopmental sequelae associated with neonatal MG. Complete resolution without long-term sequelae is expected in many cases, but the increased risk of autoimmune diseases in children born to mothers with MG necessitates ongoing awareness [9].

## Conclusions

This case demonstrates the importance of early recognition of TNMG through a comprehensive maternal history, the value of supportive care with NICU-level monitoring, and the generally favorable prognosis when the condition is identified and managed promptly. Clinicians should maintain a high index of suspicion for TNMG in any neonate presenting with feeding difficulties and hypotonia. With appropriate recognition and supportive care, the prognosis for infants with TNMG is excellent, with complete resolution expected in many cases.
